# *Notes from the Field:* SARS-CoV-2 Transmission Associated with High School Football Team Members — Florida, September–October 2020

**DOI:** 10.15585/mmwr.mm7011a3

**Published:** 2021-03-19

**Authors:** Molly Siegel, Bernhard Kloppenburg, Samantha Woerle, Scott Sjoblom, Gregory Danyluk

**Affiliations:** 1 Florida Department of Health, Polk County, Bartow, Florida.

On September 23, 2020, administrators from a Florida high school were notified of a confirmed COVID-19* case in a player on the school's football team (the index patient). The administrators informed the Florida Department of Health (FDOH), which determined that all other team members (49 players and four coaches) should be quarantined^†^ because of close contact^§^ with the index patient. By September 26, 2020, FDOH was notified of six additional team members with COVID-19 who were linked to the index patient by contact tracing.^¶^FDOH assessed the extent of transmission of SARS-CoV-2, the virus that causes COVID-19, among team members who had received positive SARS-CoV-2 test results as of September 26 and on October 6, conducted a school environmental assessment** to identify factors that might have contributed to transmission. Through a review of case reports received from health care providers and interviews with close contacts, FDOH identified 19 COVID-19 patients linked to the team, including 14 team members (12 of 50 players and two of four coaches), two nonplayer classroom contacts, and three household contacts of other team members; 18 cases were confirmed, and one was classified as probable. Thirty-one of 50 players and one of four coaches did not have test results available for review. Because the investigation was deemed a public health response, approval by the FDOH Institutional Review Board was not required.

Among the 14 team members with COVID-19, seven were symptomatic^††^; among these patients, the first onset date was September 17 (Figure). During the preceding 2 weeks, the team had held afternoon practices Monday through Thursday. Practices included outdoor exercise drills, scrimmages, play run-throughs, and hydration breaks and indoor film reviews and strength conditioning. The team played against opposing teams on September 11, 17, and 18. Mask use was infrequent during practice, and masks were not worn when playing other teams. No players from opposing teams were known to have COVID-19. The 14-day incidence was 163 cases per 100,000 persons within the school zone population,^§§^ compared with 199 within the county (*1*). Because of potential close contact between team members with COVID-19 and classmates, 267 students at the football team’s school were quarantined, resulting in approximately 2,243 person-days of lost in-person learning.^¶¶^

**FIGURE:**
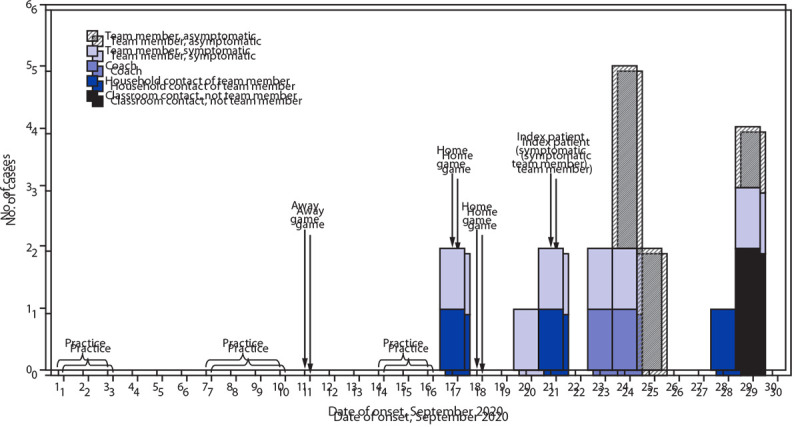
Transmission of SARS-CoV-2 among persons associated with a high school football team,* by date of onset^†^ — Florida, September 2020 * A total of 19 persons with COVID-19 who were linked to the team, including 12 players, two coaches, two classmate contacts who were not team members, and three household contacts of the team members. This included persons who received a positive SARS-CoV-2 test result from reverse transcription–polymerase chain reaction or who met the probable symptomatic case definition for COVID-19 during the investigation period. ^†^ Date of symptom onset for persons with symptomatic COVID-19 and specimen collection date for persons with asymptomatic COVID-19.

Factors that likely contributed to team transmission included 1) infrequent mask use in the weight room or during practice; 2) inadequate physical distancing and air ventilation on buses transporting players (windows remained closed); 3) infrequent cleaning and disinfection of locker rooms, weight room equipment, and communal areas (e.g., hallways and bathrooms) before and after practices; and 4) insufficient sanitizing of shared hydration system drinking nozzles between uses.

SARS-CoV-2 transmission among team members likely occurred during practice. Football and other contact sports involve frequent, direct contact, as well as physical exertion that can result in heavy respiration and higher rates of emission of virus particles (*2*–*4*). FDOH recommended that the school address the identified factors likely contributing to transmission and that the football team conduct nonphysical activities (e.g., play reviews) virtually rather than in-person. The school prevented additional exposures among staff members and students by quarantining the football team after being notified of the first player with COVID-19.

The findings in this report are subject to at least two limitations. First, testing was voluntary; not all players and classroom close contacts sought testing during their quarantine. Second, some asymptomatic persons with COVID-19 might not have been identified; therefore, the extent of SARS-CoV-2 transmission might have been underestimated.

To prevent school transmission of SARS-CoV-2 and lost quarantine-related in-person school days, school sports teams should implement recommended CDC strategies to prevent spread of COVID-19, including maintaining a distance of ≥6 ft between persons, routine mask use during practice, and testing to identify asymptomatic infected players and staff members.*** Schools should also limit extracurricular activities, including in-person sports, to minimize risk for transmission in schools and protect in-person learning as part of their mitigation strategy.^†††^

## Footnotes

* According to the Florida Department of Health, a confirmed case was defined as receipt of a positive SARS-CoV-2 reverse transcription–polymerase chain reaction (RT-PCR) test result. A probable case was defined as the occurrence of signs and symptoms of COVID-19 (e.g., cough, dyspnea, fever, myalgia, or headache) in a person who had close contact with someone who had received a positive SARS-CoV-2 RT-PCR result in the 14 days preceding illness onset during the investigation period. http://www.floridahealth.gov/diseases-and-conditions/disease-reporting-and-management/disease-reporting-and-surveillance/_documents/covid-19-case-definition.pdf

† At the time, asymptomatic close contacts were advised to quarantine for 14 days from the date of last exposure to a patient with a confirmed or probable case of COVID-19. Close contacts who developed symptoms within the 14-day quarantine period were advised to seek medical care and testing. If the symptomatic contact received a negative test result by RT-PCR for SARS-CoV-2, the person was advised that he or she could return to school and activities after the 14-day quarantine period and symptoms had resolved. Contacts who tested positive were advised to isolate for 10 days.

§ Close contact was defined as being ≥6 ft of a person with confirmed or probable COVID-19 for 15 minutes or longer within 24 hours beginning 2 days before the person became symptomatic or 2 days before specimen collection for asymptomatic persons.

¶ Contact tracing included reviewing classroom seating charts to determine which students had come into close contact with team members who had tested positive for COVID-19. If a class did not have a seating chart, the entire class was quarantined.

** The environmental assessment was based on a modified infection control assessment and response for nursing homes from CDC (https://www.cdc.gov/coronavirus/2019-ncov/hcp/assessment-tool-for-nursing-homes.html). Various areas of the school were assessed for transmission, including practice areas, buses, weight room, locker room, classrooms, bathrooms, and common areas. Interviews with students and staff members confirmed and explained how these areas were used.

††
https://www.cdc.gov/coronavirus/2019-ncov/symptoms-testing/symptoms.html

§§ The school zone is the geographic boundary that the school district uses in determining which students should attend a particular school based on their home address. Incidence was calculated using the total population of residents living within that boundary as the denominator, and the number of cases identified within the school zone (using geographic information system software to geocode reported cases by address) during September 17–30, as the numerator.

¶¶ The school reported that 51% of their high school students were in-person learners. Using the date of specimen collection for patients and exposure date for contacts, the number of missed days of school was calculated, accounting for weekends. Patients isolated for 10 days and contacts quarantined for 14 days. The number of missed school days for patients and contacts was summed and multiplied by the percentage of in-person learners (51%) to arrive at the estimated number of lost in-person school days.

***
https://www.cdc.gov/coronavirus/2019-ncov/community/schools-childcare/schools.html

†††
https://www.cdc.gov/coronavirus/2019-ncov/community/schools-childcare/operation-strategy.html
